# Carnosol ameliorated cancer cachexia-associated myotube atrophy by targeting P5CS and its downstream pathways

**DOI:** 10.3389/fphar.2023.1291194

**Published:** 2024-01-05

**Authors:** Qiao-Yu Fang, Yue-Ping Wang, Rui-Qin Zhang, Meng Fan, Li-Xing Feng, Xiao-Dong Guo, Chun-Ru Cheng, Xiong-Wen Zhang, Xuan Liu

**Affiliations:** ^1^ Institute of Interdisciplinary Integrative Medicine Research, Shanghai University of Traditional Chinese Medicine, Shanghai, China; ^2^ Shanghai Engineering Research Center of Molecular Therapeutics and New Drug Development, School of Chemistry and Molecular Engineering, East China Normal University, Shanghai, China; ^3^ Shanghai Majorbio Bio-Pharm Technology Co., Ltd., Shanghai, China; ^4^ Department of Oncology, Yueyang Hospital of Integrated Traditional Chinese and Western Medicine, Shanghai University of Traditional Chinese Medicine, Shanghai, China; ^5^ School of Chemical Engineering, Sichuan University of Science and Engineering, Zigong, Sichuan, China

**Keywords:** carnosol, cancer cachexia, myotube atrophy, proteomics, target protein, aldehyde dehydrogenase family 18 member A, P5CS

## Abstract

**Introduction:** Carnosol exhibited ameliorating effects on muscle atrophy of mice developed cancer cachexia in our previous research.

**Method:** Here, the ameliorating effects of carnosol on the C2C12 myotube atrophy result from simulated cancer cachexia injury, the conditioned medium of the C26 tumor cells or the LLC tumor cells, were observed. To clarify the mechanisms of carnosol, the possible direct target proteins of carnosol were searched using DARTS (drug affinity responsive target stability) assay and then confirmed using CETSA (cellular thermal shift assay). Furthermore, proteomic analysis was used to search its possible indirect target proteins by comparing the protein expression profiles of C2C12 myotubes under treatment of C26 medium, with or without the presence of carnosol. The signal network between the direct and indirect target proteins of carnosol was then constructed.

**Results:** Our results showed that, Delta-1-pyrroline-5-carboxylate synthase (P5CS) might be the direct target protein of carnosol in myotubes. The influence of carnosol on amino acid metabolism downstream of P5CS was confirmed. Carnosol could upregulate the expression of proteins related to glutathione metabolism, anti-oxidant system, and heat shock response. Knockdown of P5CS could also ameliorate myotube atrophy and further enhance the ameliorating effects of carnosol.

**Discussion:** These results suggested that carnosol might ameliorate cancer cachexia-associated myotube atrophy by targeting P5CS and its downstream pathways.

## 1 Introduction

Cancer cachexia is a multi-organ and systematic metabolic wasting disease characterized by involuntary loss of body weight, anorexia, and fatigue. The most important characteristic of cancer cachexia is the waste of skeletal muscle, which results in dysfunctional disease and reduced quality of life (Baracos et al., 2018; Martin et al., 2023; Setiawan et al., 2023). Approximately 50% of patients with advanced cancer would develop cancer cachexia in their late stage and cancer cachexia bears responsibility for approximately 30% of cancer-related deaths. However, in the clinical settings, there are currently not enough effective treatment options. The only drug approved for the treatment of cancer cachexia is an endogenous ligand, anamorelin hydrochloride, for the growth hormone release-promoting factor receptor (Garcia-Castillo et al., 2023; Watanabe and Oshima, 2023). To be noted, the treatment of cancer cachexia with herbal medicine has developed rapidly in recent years, and an increased number of active components in herbal medicine had been found to be potential anti-cachexia agents (Shankar et al., 2021; Han et al., 2023). In our previous study, a bioactive diterpene compound which was found in Lamiaceae spp., carnosol (CS), performed preferable mitigating effects on cancer cachexia-associated muscle atrophy in C26 cancer cachexia model mice (Lu et al., 2021). We have applied and have been authorized the national patents of China for the usage of CS as well its analogs in the treatment of cancer cachexia (Patent No. CN113244221B, CN113244222B).

Though we had found the effects of carnosol such as the inhibition on the NF-κB signaling pathway in ameliorating cancer cachexia-associated muscle atrophy (Lu et al., 2021), the mechanisms of carnosol still need further clarification. The target-related proteins of carnosol in muscle cells, neither direct nor indirect target-related proteins, had not been reported before. In previous reports, carnosol had been reported to have several kinds of pharmacological effects, which includes antioxidant, anti-inflammatory, anti-cancer, and protecting effects on cells against different injuries (Johnson, 2011; Yao et al., 2014; Shi et al., 2020; Kalantar et al., 2021; Chen et al., 2022b; Ren et al., 2022; Wu et al., 2022; Ji et al., 2023). For example, lung damage in rats caused by bleomycin can be ameliorated by carnosol through bringing down oxidative stress and inflammation (Kalantar et al., 2021). Carnosol could protect human retinal microvascular endothelial cells against oxidative stress injury via Nrf2-related pathways (Ren et al., 2022). Besides, carnosol could ameliorate neurodegenerative diseases via improving proteostasis and ameliorating mitochondrial disorders (Chen et al., 2022b). Furthermore, carnosol has also been reported to moderate non-alcoholic fatty liver disease by targeting PRDX3 to inhibit mitochondrial disorder and apoptosis (Geng et al., 2021). These results suggested that both direct anti-oxidant activity and in-direct regulation of oxidative stress response (Lian et al., 2010; Kalantar et al., 2021; Karagianni et al., 2022; Ji et al., 2023) might play important roles in the effects of carnosol. Furthermore, regulation of stress-related pathways such as heat shock systems (Mohebati et al., 2012; Shi et al., 2020) and inhibition of inflammatory pathways such as the NF-κB pathway (Lo et al., 2002; Lian et al., 2010; Yao et al., 2014; Li et al., 2021) were also reported to be included in the effects of carnosol.

In the present study, to further clarify the mechanisms of carnosol in ameliorating cancer cachexia-associated muscle atrophy, possible direct target proteins of carnosol were searched using DARTS (drug affinity-responsive target stability) assay while possible indirect target proteins of carnosol in C2C12 myotubes were searched using proteomic analysis. Furthermore, the signal network including both the indirect and direct targets of carnosol was constructed. The involvement of the important target protein of carnosol such as P5CS (Aldh18a1) was then confirmed. P5CS (Aldh18a1) is linked to antioxidant function and amino acid metabolism. The P5CS (Aldh18a1) regulates glutamate replenishment, which affects the metabolism of glutamine and glutathione (Colonna et al., 2023). When glutamate-cysteine ligase is overused, it can result in a glutamate shortage, which interference the synthesis of amino acids, including ornithine and proline. To be noticed, amino acids are crucial for the oxidation and redox metabolism. For instance, leucine can stimulate the mTORC1 pathway, keeping the accumulation of proteins (Beaudry and Law, 2022). Leu’s metabolism product may lower muscle oxidation and maintain protein catabolism and anabolism, while arginine and glycine can maintain the balance of glutathione and immune function. The combination treatment of Leu, Ile, and Val may reduce oxidation stress and increase the survival rate (Ragni et al., 2022). As reported by previous research, the levels of glutamine and branched-chain amino acids in patients with pancreatic cancer cachexia significantly decreased (Tuma et al., 2021), and reduced quantities of amino acids and glutathione may be caused by cancer cachexia (Hack et al., 1996; Droge et al., 1997; Droge et al., 1998; Li et al., 2023), Additionally, glutamine plays an important role during cancer cachexia since it can be depleted from skeletal muscle by immunological cells, neuroendocrine organs, and immune system activities (de Blaauw et al., 1999). Carnosol has been reported to improve the antioxidant function of glutathione (Singletary, 1996; Takahashi et al., 2009; Chen et al., 2011), therefore, we further checked the effects of CS on glutathione synthesis, glutathione metabolism, antioxidant system and amino acid metabolism whether related to the atrophy ameliorating effect of CS in cancer cachexia model in the present study.

## 2 Materials and methods

### 2.1 Reagents

Penicillin/streptomycin, DMEM medium (high glucose), RPMI-1640 medium, and trypsin/EDTA were provided by Hyclone (Los Angeles, CA, United States). Carnosol was provided by NatureStandard Co., LTD. (Shanghai, P.R. China). FBS was purchased from Biological Industries (Kibbutz Beit Haemek, Israel). Halt Protease, and Phosphatase Inhibitor Cocktail (×100) and RIPA Lysis were obtained from Thermo Scientific (Rockford, IL, United States). DAPI and BCA protein quantification kit were purchased from Beyotime (Hangzhou, P.R. China). And most of the other chemicals were provided by Sigma-Aldrich Chemical Co., (St. Louis, MO, United States).

### 2.2 Cell culture

Murine C2C12 (ATCC, Manassas, United States) myoblasts were cultivated in high-glucose DMEM medium with 10% (v/v) fetal bovine serum. To obtain C2C12 myotubes, DMEM with 2% horse serum was implied to induce the differentiation of C2C12 myoblasts, and the multinuclear myotubes form in 3–5 days. C26 murine colon adenocarcinoma cells and LLC murine lung adenocarcinoma cells were provided by SIMM (Shanghai, China) and ATCC (Manassas, United States), while RPMI-1640 and high-glucose DMEM [both with 10% (v/v) FBS] were used to culture these 2 cells respectively. Rat H9c2 cardiomyocytes (Cell bank of the Institute of Biochemistry and Cell Biology, Shanghai, China) were cultured in high-glucose DMEM with 10% (v/v) FBS. The environment of the incubator is 5% CO_2_, at 37°C.

### 2.3 *In vitro* cancer cachexia myotube atrophy model

The cancer cachexia model was established using atrophied C2C12 myotube, and the detailed method was described in our previous reports (Shen et al., 2022; Fan et al., 2022; Lu et al., 2021). To obtain the conditioned medium of tumor cells, C26 colon adenocarcinoma cells or LLC lung adenocarcinoma cells, the medium was replaced with fresh DMEM medium after 2 days of culture, then collect the medium and separate the cell fragments by centrifugation (3,000 ×g, 10 min at 4°C) after 48 h. The collected conditioned medium can be used promptly or keep in −80°C, and the concentration for myotube atrophy was from 30% to 50%, which was mixed with fresh medium. Briefly, C2C12 myotube atrophy was generated by C26 conditioned medium or LLC conditioned medium after 5 days of differentiation. At the day 5 of differentiation, most of the C2C12 myocytes had differentiated into myotubes, and the CS (carnosol) or 0.1% DMSO (solvent control) were treated together with the conditioned medium of tumor cells. The effects of CS at different doses (10, 15, 20, 25 μM) for 48 h or the effects of 20 μM CS for different time periods (36, 48 or 72 h) were observed. Then, cells were stained with H&E staining and the images were taken by Cytation 5 (BioTek, United States). As the method represented in the previous reports of our team, Digimizer software was applied to measure the diameter of the myotube (Lu et al., 2021; Fan et al., 2022; Shen et al., 2022), the diameter of three different parts of the muscle tube were quantified and at least 100 myotubes of each group were measured randomly. Three repeated experiments were conducted independently and the results were statistically analyzed.

### 2.4 DARTS analysis

To identify the direct target proteins of carnosol, “DARTS” (drug affinity-responsive target stability), a micro-chemical-compound target-finding strategy was applied as represented previously. Since the possible binding proteins are less susceptible to degradation during proteolysis, the target has a higher detection ratio in treatment group compared to control group in proteomic analysis (Qu et al., 2016; Wang et al., 2022). Briefly, 1 × 10^7^ C2C12 myotubes were lysed in M-PER (Pierce, Thermo Scientific, United States) buffer containing protease inhibitors and then centrifuged. The resulting lysate was divided into two tubes and incubated with either 20 μL DMSO (as a solvent control) or 20 μM carnosol after the addition of 10 TNC buffer. From each sample, 200 mg of protein was processed with trypsin (Promega, United States) digestion and quantified with the BCA Protein Assay Kit (Thermo Scientific, United States). Peptides were isolated and analyzed with an Easy-nLC 1,200 system linked to a Q Exactive HF (Thermo Scientific, United States). MaxQuant 1.6.5.0 was used to process and search the raw data, with database searches using the MaxQuant proteomics contaminants database and the UniProt mouse protein database (release 2022_11). The reversed database searches were adopted to assess the false discovery rate of peptide and protein (FDR). The Student’s *t*-test was used to assess the statistical significance of the difference in protein abundance between carnosol and control samples. *p* < 0.05 was regarded as statistically significant. Results with a fold change of more than a 1.2-fold increase between carnosol and DMSO were displayed.

### 2.5 Molecular docking

According to our earlier report (Wang et al., 2023), molecular docking was carried out. Briefly, PubChem provided the structure document of the carnosol, and the Alpha Fold Protein Structure Database provided the simulated structure of the protein P5CS. To predict the binding score between the small molecule CS and the protein ALDH18A1, the structures of CS and ALDH18A1 were downloaded from the Pubchem database (Kim et al., 2023), and the Alphafold protein structure database (Armstrong DR et al., 2020), respectively. The software Sailvina (Final Version 1.0) (beikwx, 2020) was used to prepare the docking sites of CS and ALDH18A1, followed by molecular docking and extraction of docking fractions. Then, the software Pymol (Version 2.3.4) was used for molecular docking visualization, and the PLIP website was used to predict docking sites. The molecular binding process could predict hydrogen bonds, hydrophobic interactions, and energy changes, and the molecular docking fraction could reflect the interaction between Aldh18a1 and carnosol to a certain extent, according to the comprehensive evaluation of a number of indicators. The smaller the binding score in molecular docking, the stronger the binding affinity. Generally, binding score less than −7 indicates that there is a binding affinity between the small molecule and the protein.

### 2.6 Cellular thermal shift assay (CETSA)

The potential for carnosol to interact with P5CS in cells was confirmed in both C2C12 myotubes and H9c2 cardiomyocytes using CETSA as reported by our team previously (Wang et al., 2023). For C2C12 cells, cells were treated with carnosol directly. Briefly, cells were cultivated in 10 cm cell culture dishes and exposed to 1 h treatment of 20 μM carnosol or the same amount of DMSO (solvent control). After washing with PBS, the cells were digested and collected, and the protein was extracted using NP40 lysis buffer. For H9c2 cells, cellular proteins were extracted from cells and then incubated with 20 μM carnosol or the same amount of DMSO (solvent control) for 30 min on a rotator in room temperature, followed by incubating for 1 h at 4°C. Each group of cell lysate protein samples was divided into six aliquots, then the ProFlex PCR System (ThermoFisher Scientific, United States) was employed to heat samples at the specified temperature for 5 min, rest in 26°C for 3 min, and centrifuged at 12,000 rpm for 10 min at 4°C. Finally, the P5CS in the supernatant was detected by Western blotting analysis. For internal comparison, the β-Actin protein was used. The quantitative analysis of the protein expression in each group was performed with ImageJ. Three replication experiments were performed and the results were used for statistical analysis.

### 2.7 Proteomic analysis

“Shotgun proteomics based on liquid chromatography-high resolution tandem mass spectrometry” was applied as stated in our previous research (Feng et al., 2021) to check the protein expression profiles of C2C12 myotubes in the control group (treated with solvent control 0.1% DMSO for 48 h), C26 medium group (treated with C26 conditioned medium for 48 h), CS group (treated with carnosol at 20 μM for 48 h) and C26+CS group (treated both with C26 conditioned medium and 20 μM carnosol for 48 h). Samples of three independent experiments were collected. For sample collection, cells were dissolved in 8 M urea with 1% SDS after washed with PBS for twice. Cell proteins were broken down into peptides and collected after trypsin (Promega, United States) digestion for a whole night. To measure multiple samples simultaneously in the same study, TMT 16-plex^pro^ reagents (Thermo Scientific, United States) were adopted to label peptides of each sample. Following the manufacturer’s instructions, different samples of the control group, C26 medium group, CS group and C26+CS group were labeled with isobaric compounds. Thermo Fisher Scientific’s Orbitrap Exploris 480 mass spectrometer was used to acquire mass spectrometric data together with the company’s EASY-nLCTM 1200 UHPLC system. Proteome Discoverer (v2.5) was used to process the raw data, and the Mascot (Matrix Science, London, United Kingdom; version 2.2) engine was applied for the search. KEGG and GO analyzed DEPs which upregulated (FC > 1.2) or downregulated (FC < 0.83) between two groups. In the volcano plot, DEPs which Log2FC > 0.25 or < −0.25 were marked as significant changed proteins. The proteomics data have been uploaded to the ProteomeXchange Consortium (Ma et al., 2019) via the iProX partner repository (Sirnio et al., 2019; Chen et al., 2022a). The dataset identifier is PXD043464. The Login ID is Qiaoyu_Fang, and the password is 987654321.

### 2.8 Bioinformatic analysis and establishment of interaction network

Bioinformatic analysis was conducted by utilizing the Majorbio Cloud Platform. The analysis of the GO and KEGG pathway enrichment for the differentiated proteins and prospective targets were plotted. Utilizing Venny 2.1.0, it was possible to distinguish the proteins significantly changed and the prospective targets of carnosol in myotubes. Protein-protein interaction (PPI) analysis conducted by the STRING database was used (Szklarczyk et al., 2019) to establish the interaction network between the direct and indirect target proteins of carnosol.

### 2.9 Western blotting assay

Western blotting assay was performed as our previous description (Lu et al., 2021; Fan et al., 2022; Shen et al., 2022). Briefly, cells were lysed using RIPA buffer, and the lysates were centrifuged at 12,000 rpm at 4°C for 30 min. The BCA Protein Assay Kit was employed to quantify the protein concentration (Beyotime, Shanghai, China). For Western blotting analysis, same amounts of samples were separated by 12.5% SDS-PAGE gel electrophoresis and transferred to a PVDF membrane. After incubation with 5% skim milk for 2 h, the membrane was then incubated at 4°C overnight with the corresponding primary antibody. The primary antibodies used in the present study are P5CS (P5CS Rabbit PolyAb, Proteintech, 17719-1-AP, #00108767, 1:2,000) and β-actin (β-actin (C4), Santa Cruz, sc-47778, #J1119, 1:2,000). The membranes were then incubated with HRP-conjugated secondary antibodies, HRP-conjugated goat anti-rabbit secondary antibody (Zen Bio, 511203, M09FE73, 1: 5,000) for P5CS and HRP-conjugated goat anti-mouse secondary antibody (Zen Bio, 511103, #L25DE51, 1: 5,000) for β-actin, and incubate for 1 h. Electrochemiluminescence (ECL) reagents (Thermo Fisher, United States) were used to identify the membranes, and ChemiDoc imaging equipment was used to capture the image. Using ImageJ software, a quantitative analysis of the protein expression levels was performed.

### 2.10 Amino acid profile analysis

The content of free amino acids in C2C12 myotubes was quantified with an LC-MS/MS (UHPLC-Qtrap) system, which is optimized based on reported methods (Yuan et al., 2021). Briefly, samples were mixed with water and 0.15% DOC, vortexed, and mixed with an internal standard solution (Lys-d4/Try-d5/Gln-d4, 100 μg/mL). The mixture was sonicated for 10 min (5°C, 40 kHz), and 10 M trichloroacetic acid (TCA) was added. Samples were frozen for 10 min, then centrifuged (14,000 rcf, 4°C, 10 min) and mixed with water and vortexed, and the mixture was filtered with a 0.2 μm PTFE filter membrane (Biotage, Sweden). EASY-nLCTM 1200 UHPLC (Thermo Scientific, United States) with AdvanceBio MS Spent Media (2.1 × 50 mm, 2.7 µm) (Agilent, United States) were employed for the chromatography, the column temperature was 40°C and injection volume was 1 μL. As the mass spectrometry method reported (Ma et al., 2022), for the qualitative and quantitative determination of the target in the samples, ESI-MS/MS with the MRM mode was conducted by SCIEX QTRAP 6500+ (AB SCIEX, Foster City, CA, United States). AB Sciex quantitative software OS automatically identifies and integrates each ion fragment using default parameters, with manual inspection assisting the process.

### 2.11 Knockdown expression of P5CS by siRNA

The siRNA oligos for Aldh18a1 (the gene encoding P5CS) and the negative control siRNAs were provided by the Sangon Biotech Company (Shanghai, P.R. China). The amount of siRNA treated for each well was 20 μM, and 10 pMol. The siRNA sequences for Aldh18a1 are presented:

siAldh18a1-445: sense (5′-3′) CGC​CAA​GAG​AAU​UGU​AGU​GAA​TT

siAldh18a1-897: sense (5′-3′) CCC​AUC​GUC​AAC​ACA​AAC​GAU​TT

siAldh18a1-2586: sense (5′-3′) CUC​AAG​UAU​CUU​CAC​GAG​AAU​TT.

Transfection of siRNAs (Aldh18a1 siRNA or negative siRNA) into the C2C12 myotubes was conducted using Lipofectamine RNAiMAX (Invitrogen, Carlsbad, United States) as described in our previous report (Wang et al., 2023).

### 2.12 Microscale thermophoresis (MST)

A plasmid carrying the GFP target protein was purchased from Guannan Biology (Hangzhou, China), and the sequence information of target protein was obtained from NCBI, the gene name is: Aldh18a1 aldehyde dehydrogenase 18 family, member A1 [*Mus musculus* (house mouse)], and the Gene ID is 56,454. To verify the ability of carnosol to bind P5CS, the plasmid was expressed in 293T cells. After 48 h, the cell lysate is collected with NP40 cell lysate buffer (300 μL/10 cm plate), flicked for 3 times with 10 min interval, then centrifuged at 12,000 rpm, 4°C, 30 min for subsequent use. MonolithTM NT.115 MST equipment was applied to examine the binding affinity (Nano temper, Germany). The raw fluorescence of GFP-Aldh18a1 was limited between 600–800 counts and the primary concentration of the ligand (carnosol) was 1 mM, then the carnosol was gradient half diluted with GFP-Aldh18a1 cell lysate for 15 times. After rested for 30 min on ice, the carnosol-GFP-Aldh18a1 mixture was prepared for the observation of ligand-protein binding affinity. The mass action equation in NanoTemper software was applied to calculate the Kd value.

### 2.13 Reactive oxygen species assay

The Meilun reactive oxygen species assay kit (Meilunbio, Dalian, China) was utilized to measure the amounts of reactive oxygen species (ROS) in various groups. The kit relies on measuring the fluorescence intensity of DCFH-DA. In brief, C2C12 cells were seeded into 24 well plates and allowed to differentiate into myotubes. The myotubes were subsequently treated with C26 medium or carnosol based on their grouping. After 48 h, DCFH-DA (1:1,000 diluted with serum-free culture media) was used to stain ROS in myotubes. Following 30 min of incubation at 37°C, the DCFH-DA were removed and cells were washed three times with media, and Cytation 5 (BioTek, United States) was used to capture photos and detect the fluorescence intensity. ImageJ was utilized to perform the quantitative analysis of fluorescent intensity.

### 2.14 Statistical analysis

All values are presented as the mean ± standard error of the mean (SEM). Prism 7.0 software (Graphpad Software Inc., La Jolla, CA, United States) was applied to process data. One-way ANOVA combined with Tukey’s *post hoc* multiple comparisons was used to test the statistical significance of differences among groups, and an unpaired two-tailed Student’s *t*-test was adopted to process two-group comparison. Statistical significance was defined as a *p*-value of less than 0.05. The proteins in the volcano map and KEGG/GO analysis are differentially expressed proteins between the C26+CS group and the C26 medium group (fold change >1.2 or fold change <0.83). The proteins shown in PPI map are differentially expressed proteins between the C26+CS group and the C26 group (fold change >1.5 or fold change <0.5).

## 3 Results

### 3.1 Carnosol ameliorated C2C12 myotube atrophy and the elevated ROS level

**FIGURE 1:**
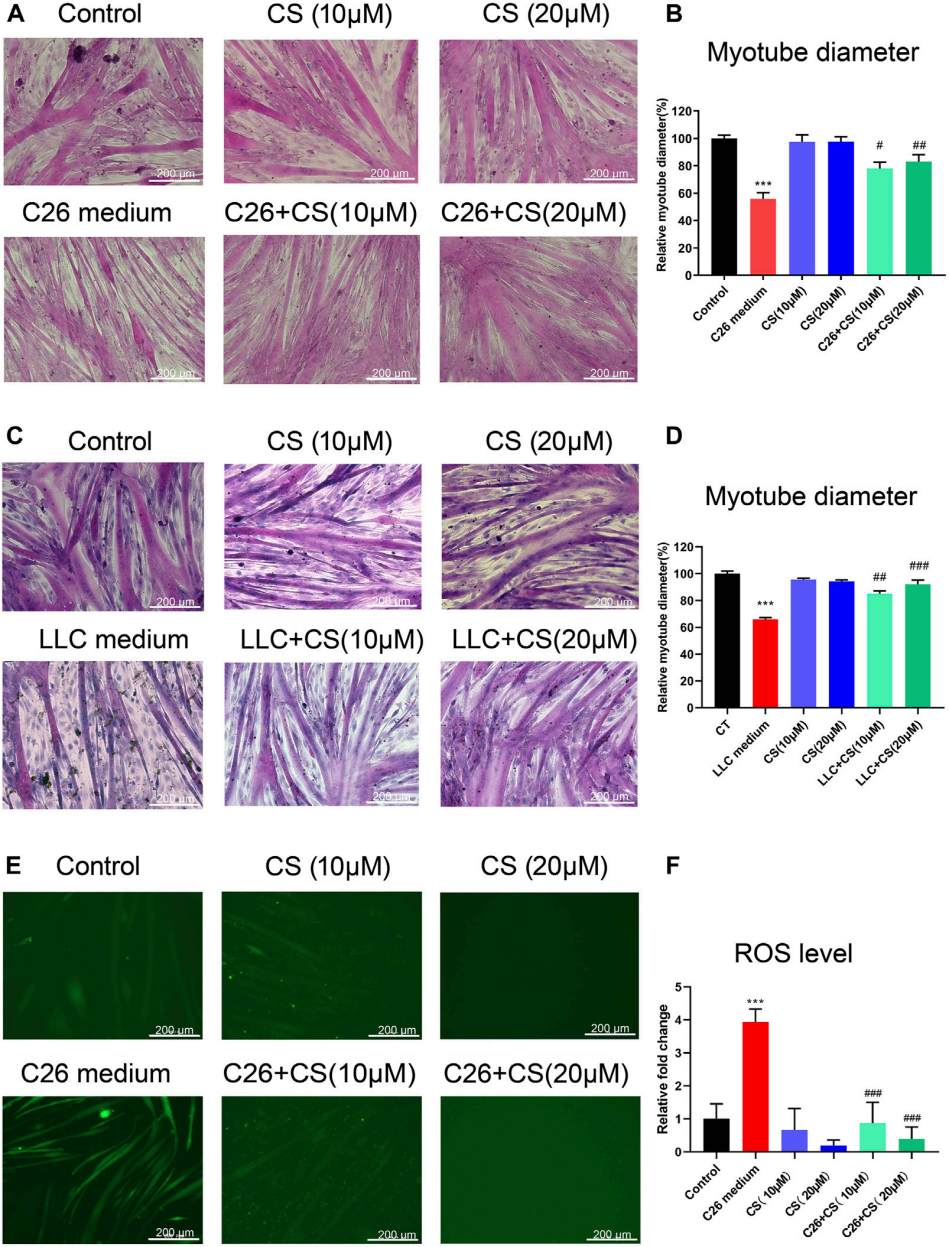
Carnosol ameliorated the myotube atrophy of C2C12 myotubes induced by the C26 tumor medium or LLC tumor medium. **(A)** Representative images of H&E staining of C2C12 myotubes underwent treatment of C26 medium with or without the presence of carnosol (CS). Scale bar, 200 μm. **(B)** Myotube diameter quantification after C26 medium treatment with or without the presence of carnosol. **(C)** Representative images of H&E staining of C2C12 myotubes underwent treatment of LLC medium with or without the presence of carnosol (CS). Scale bar, 200 μm. **(D)** Myotube diameter quantification after LLC medium treatment with or without the presence of carnosol. **(E)** The representative image of ROS fluorescence intensity of myotubes underwent treatment of C26 medium with or without the presence of carnosol (CS). Scale bar, 200 μm. **(F)** The quantification results of ROS fluorescence intensity in different groups. Data are presented as mean ± SEM (*n* = 3). ***p < 0.001 vs. control group; #*p* < 0.05, ##*p* < 0.01, ###*p* < 0.001vs. C26 medium or LLC medium group.

Figures 1A, B represented the C2C12 myotube atrophy caused by C26 medium and the dose-dependent protecting consequence of carnosol (CS). As shown in Figures 1A, B (representative results and statistical results, respectively), in contrast to the myotubes of the control group, the diameters of the myotubes treated with C26 tumor cell medium decreased significantly, suggesting that C26 medium induced myotube atrophy. While, carnosol (CS) could concentration-dependently ameliorate the C2C12 myotube atrophy caused by C26 medium. Similar results were found in C2C12 myotubes treated with LLC tumor medium. As shown in Figures 1C, D (representative results and statistical results, respectively), LLC tumor medium induced myotube atrophy while carnosol (CS) can dose-dependently ameliorate the myotube atrophy. The results suggested that carnosol could ameliorate myotube atrophy induced by simulated tumor cachexia injuries. In addition, the experiments checking the effects of Carnosol on myotube atrophy at different time points (36, 48, and 72 h) and different doses (10, 15, 20, 25, and 30 μM) were shown in the Supplementary Figures S1, S2, respectively. As shown in Supplementary Figures S1, S2, CS also could ameliorate the myotube atrophy of C2C12 myotubes induced by 36 or 72 h treatment of C26 conditioned medium, and the CS treatment at 10, 15, and 20 μM exhibited dose-dependently ameliorating effects on myotube atrophy. CS treatment at 20 μM exhibited the best ameliorating effects on myotube atrophy. However, CS treatment at dose higher than 20 μM, such as 25 μM, did not exhibit better ameliorating effects than that of 20 μM on myotube atrophy, possibly because of cytotoxicity. The results suggested that CS could exhibit considerable ameliorating effects on myotube atrophy induced by simulated cancer cachexia injury. Additionally, as shown in Figures 1E, F, ROS level of C2C12 myotubes under C26 TCM induction significantly increased while CS treatment could ameliorate the elevation of the ROS level, which exhibited the ROS eliminating effect of carnosol in C2C12 myotubes underwent injury.

### 3.2 Proteomic analysis results revealed the proteins involved in the atrophy of myotubes and the ameliorating effects of carnosol

**FIGURE 2:**
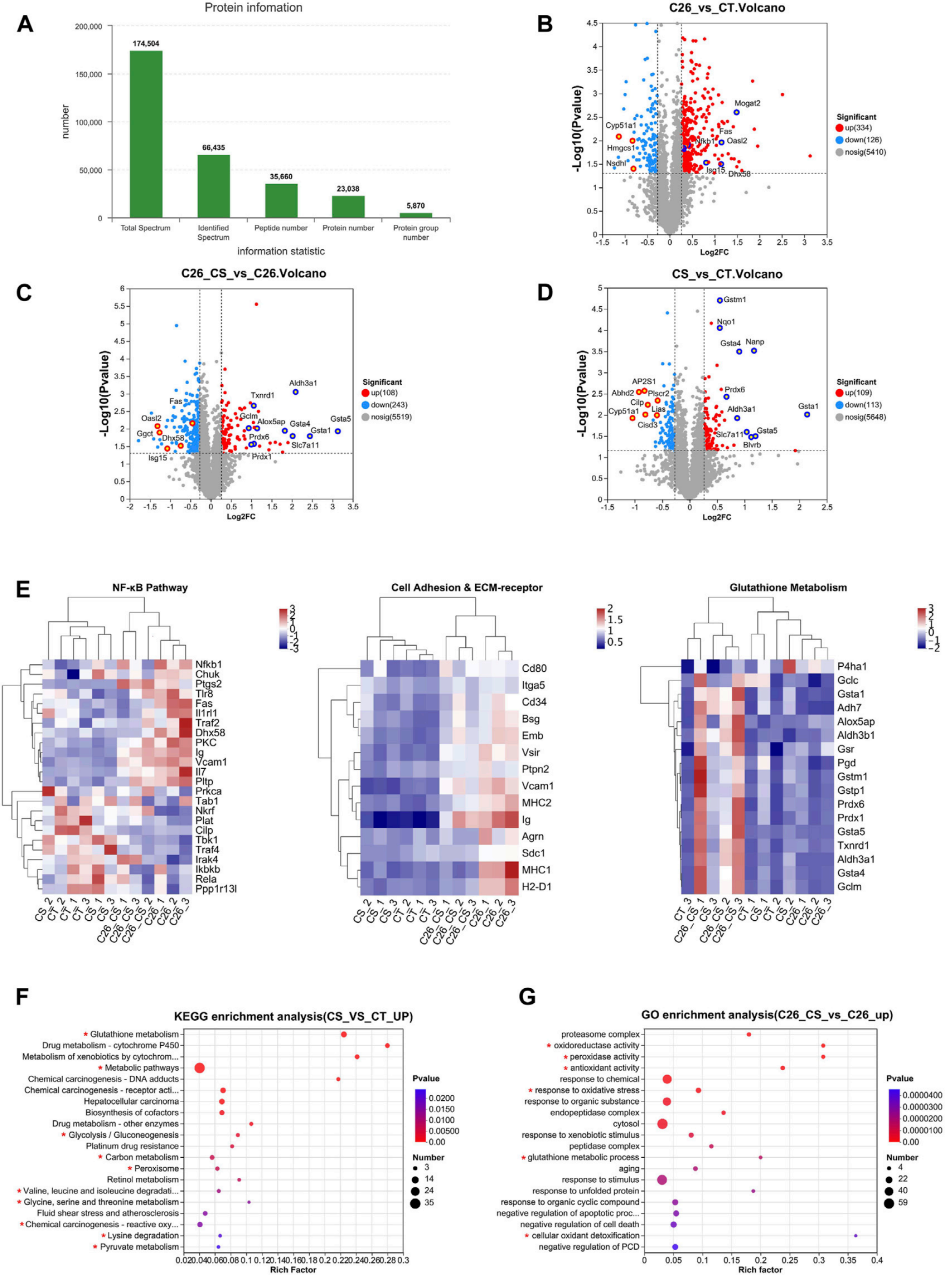
Proteomic analysis of C2C12 myotubes showed the difference in protein expression profiles of the CT group (Control), C26 group (C26 medium model), CS group (carnosol at 20 μM) and C26+CS group (C26 medium together with carnosol at 20 μM). **(A)** The overall protein numbers and protein groups (proteins appear in all four groups) were detected in global proteome analysis. **(B)** Volcano plots of the distribution of the differentially expressed proteins (DEPs) in the C26 group compared to the CT group. **(C)** Volcano plots of the distribution of the DEPs in the C26+CS group compared to the C26 group. **(D)** Volcano plots of the distribution of the DEPs in the CS group compared to the CT group. **(E)** Heat map of NF-κB pathway, cell adhesion and ECM-receptor pathway and glutathione pathway. **(F)** KEGG analysis of DEPs which upregulated (FC > 1.2) in the CS group compared to the CT group. **(G)** GO analysis of DEPs which upregulated (FC > 1.2) in the C26+CS group compared to the C26 group. In the volcano plot, *Y*-axis represents -Log10 *p*-value and *x*-axis represents the Log2 ratio (C26/CT), and the vertical dashed lines indicate the differential threshold limit (Log2FC > 0.25 or < -0.25), the horizontal dashed lines show significant threshold limit [-log10 (*p*-value) >1.3]. Pearson correlative analysis was employed for the hierarchical clustering of the heat maps. In KEGG and GO analysis, the *x*-axis represents the enrichment rate, calculated as Ratio in study/Ratio in pop; the *y*-axis represents -log10 (*p*-value), and the default parameter is *p*-value uncorrected. The size of the bubble is proportional to the number of proteins in the protein set enriched by the GO Term. The different colors of the bubbles represent the *p*-value. The KEGG pathways and GO functions are marked with red asterisks are significantly changed groups.

To identify the proteins changed during C2C12 myotube atrophy result from C26 tumor medium treatment and the ameliorating effects of carnosol, we performed high-resolution mass spectrometry to analyze C2C12 myotube samples in control group, C26 medium group, CS group, and C26+CS group. In general, there are altogether 23,038 proteins were identified from all C2C12 myotube samples and approximately 5,870 protein groups were identified in the four groups (Figure 2A). 460 differentially expressed protein (DEPs) was found in the C26 medium group vs. CT group, in which 334 were upregulated and 126 were downregulated (Figure 2B), with the Fold change >1.2 or Fold change <0.38. Proteins such as Fas (Tumor necrosis factor receptor superfamily member 6), Isg15 (interferon-stimulated gene 15), and Dhx58 (DExH-Box helicase 58), which play important roles in the regulation of inflammatory response including activation of NF-κB pathway, were found to be upregulated in C26 medium group compared with the control group (Figure 2B). KEGG enrichment analyses also revealed that the upregulated proteins in the C26 vs. CT group were related to the NF-κB signaling pathway, Cell adhesion molecules, ECM-receptor interaction, etc. (Figure 2E). Results of comparing the protein expression profiles of the C26+CS group and the C26 group showed that, the number of downregulated proteins in the C26+CS group is more than the number of upregulated proteins, and proteins related to the NF-κB signaling pathway such as Fas and Isg15 were downregulated in the C26+CS group vs. the C26 group (Figure 2C). The results suggested that carnosol treatment could partly reverse the change in protein expression profiles of myotubes induced by the C26 tumor medium. Treatment of carnosol alone (CS group) induced change in the expression of 127 proteins, in which the upregulated proteins such as Peroxiredoxins (Prdx6) and Glutathione-S transferases (Gsta5, Gsta4, Gsta1) belong to the anti-oxidant system (Figure 2D). As shown in the heat maps combined with hierarchical clustering of specific GO/KEGG entries in KEGG enrichment analyses (Figure 2E), proteins upregulated in the C26 group vs. CT group which also downregulated in the C26+CS group vs. C26 group were mainly related to NF-κB pathway, cell adhesion pathway and ECM-receptor pathway, while proteins upregulated in C26+CS group vs. C26 group were mainly related to glutathione metabolism. KEGG analysis further revealed that the upregulated proteins in the CS group vs. CT group were mainly related to glutathione metabolism and amino acid metabolism pathways, such as Valine, leucine and isoleucine degradation, Glycine, serine and threonine metabolism, and Lysine degradation (Figure 2F). For proteins upregulated in the C26+CS group vs. C26 group, GO enrichment analysis results showed that carnosol treatment under C26 medium injury still upregulated the expression of proteins related to glutathione metabolism, anti-oxidant activity, response to oxidative stress, etc. (Figure 2G). These results suggested that carnosol had regulative effects on proteins mainly related to glutathione metabolism and anti-oxidant system, which might be involved in its protective effects on myotubes against injury of C26 tumor medium treatment.

### 3.3 Expression of proteins related to glutathione metabolism, antioxidant system and heat shock response were regulated by carnosol

**FIGURE 3:**
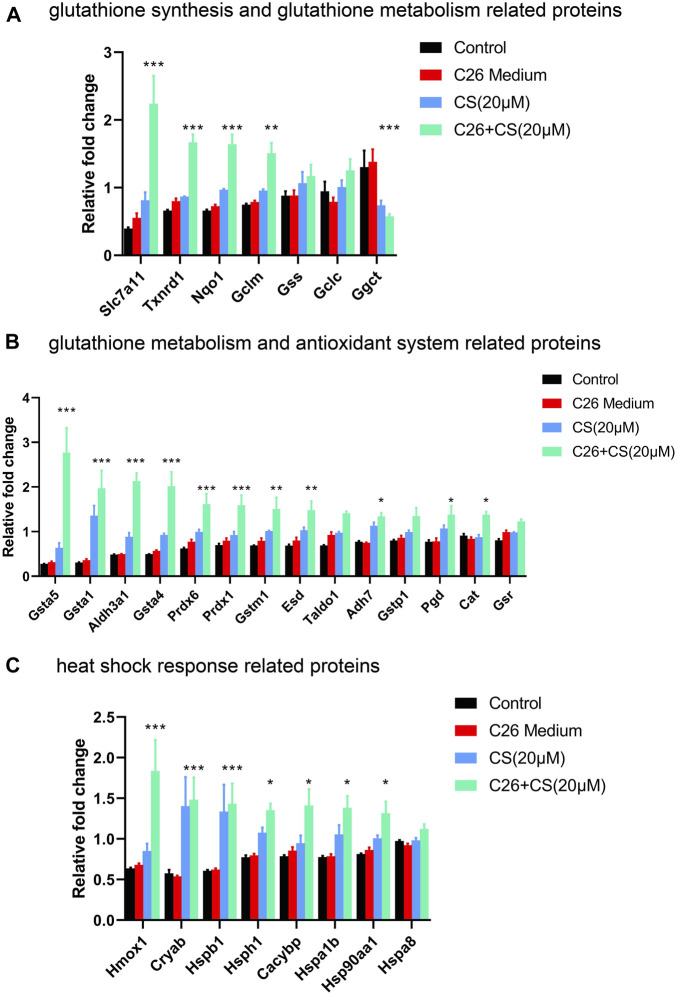
The expression levels of proteins related to glutathione synthesis and glutathione metabolism, antioxidant system and heat shock response in C2C12 myotubes of the control group, C26 group, CS group and C26+CS group. **(A)** The histogram of the expression levels of proteins related to glutathione synthesis and glutathione metabolism. **(B)** The histogram of the expression levels of proteins related to glutathione metabolism and the antioxidant system. **(C)** The histogram of the expression levels of proteins related to heat shock response. Data are presented as mean ± SEM (*n* = 3). *p < 0.05, **p < 0.01, ***p < 0.001 vs. control group.

The expression levels of the three subsets of proteins that exhibited close interaction with P5CS in C2C12 myotubes of different groups including the control group, C26 group, CS group and C26+CS group were shown in Figure 3. The individual histogram (Figure 3A) of the expression of proteins related to glutathione synthesis and glutathione metabolism showed that proteins such as Slc7a11 were upregulated while proteins such as Ggct were downregulated in the C26+CS group compared with the control group. The individual histogram (Figure 3B) of the expression of proteins related to the glutathione metabolism and antioxidant system showed that proteins such as Glutathione S-transferases (Gsta5, Gsta1, Gsta4) and Peroxiredoxins (Prdx6, Prdx1) were upregulated in C26+CS group compared with the control group. The individual histogram (Figure 3C) of the expression of proteins related to heat shock response showed that proteins such as Hspb1, Hsph1 and Hsp90aa1 were upregulated in the C26+CS group compared with the C26 group. Notably, these proteins exhibited no significant change in the C26 group vs. control group but exhibited similar change in the CS group vs. control group (Figure 3). These results further confirmed the regulative effects of carnosol treatment on these proteins, with or without the treatment of cancer cachexia injury.

### 3.4 Carnosol may regulate the amino acid disorder caused by cancer cachexia

**FIGURE 4:**
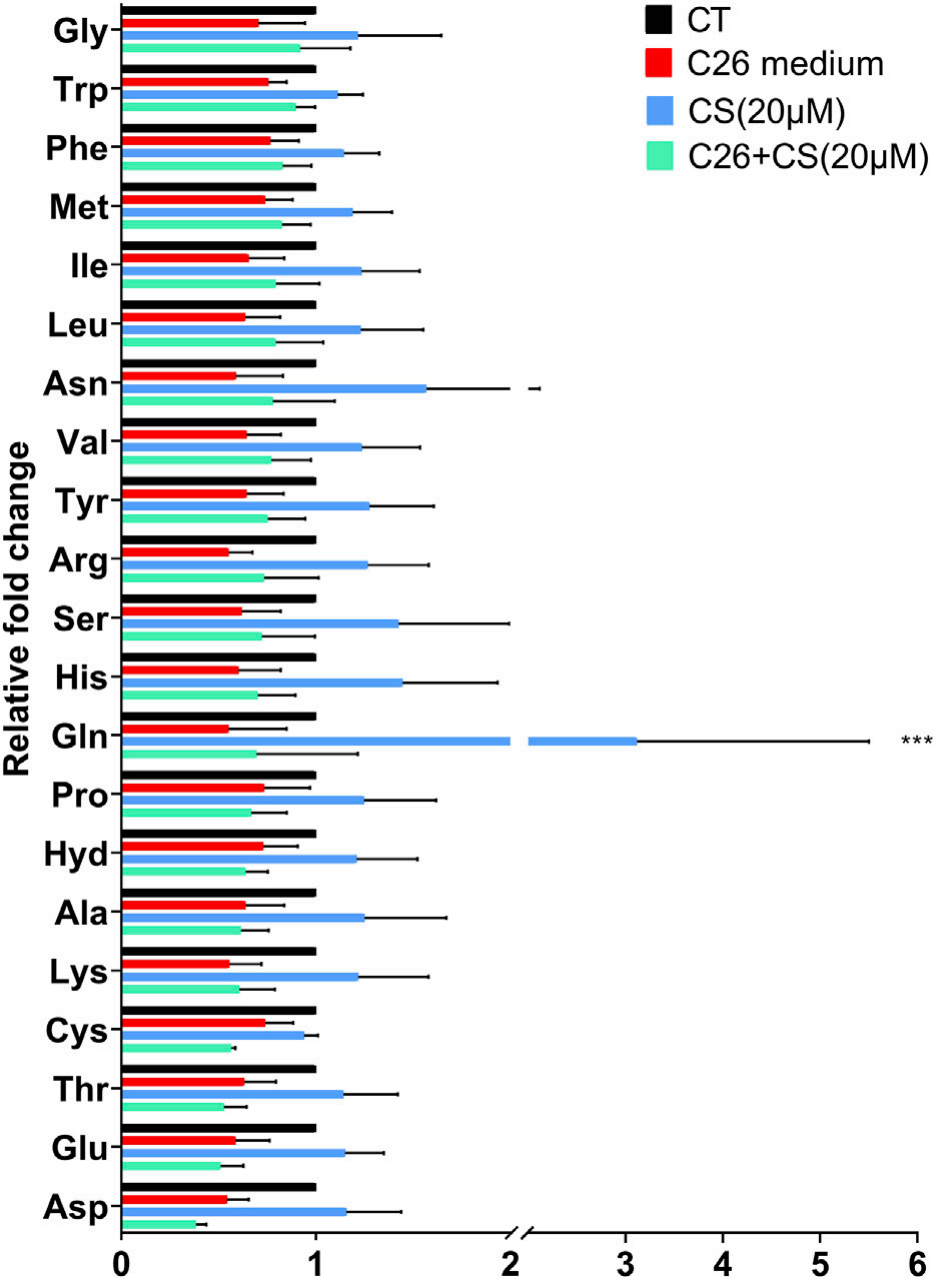
The individual histograms of 21 essential amino acid profiles of C2C12 myotubes in the CT group (Control), C26 group (C26 medium model), CS group (carnosol at 20 μM) and C26+CS group (C26 medium together with carnosol at 20 μM). ***p < 0.001 vs. control group.

Figure 6 showed the levels of 21 essential amino acids in C2C12 myotubes in different groups. The histogram in Figure 4 indicated that, generally, amino acid levels decreased in the C26 group but increased in the CS treatment group. As shown in the results of the C26+CS group, CS treatment could partly ameliorate the decrease in the amino acid levels of 14 amino acids, such as glutamine (Gln), arginine (Arg), histidine (His), glycine (Gly), and asparagine (Asp), induced by the C26 medium. Among the amino acids, glutamine exhibited to be the special one. The increase of glutamine induced by CS was significant and the fold change ratio of the CS group/CT group was 3.1. The results suggested that regulation on amino acid metabolism such as glutamine metabolism might play an important role in the effects of CS.

### 3.5 DARTS, CETSA analysis and MST assay results revealed P5CS to be the direct target protein of carnosol

**FIGURE 5:**
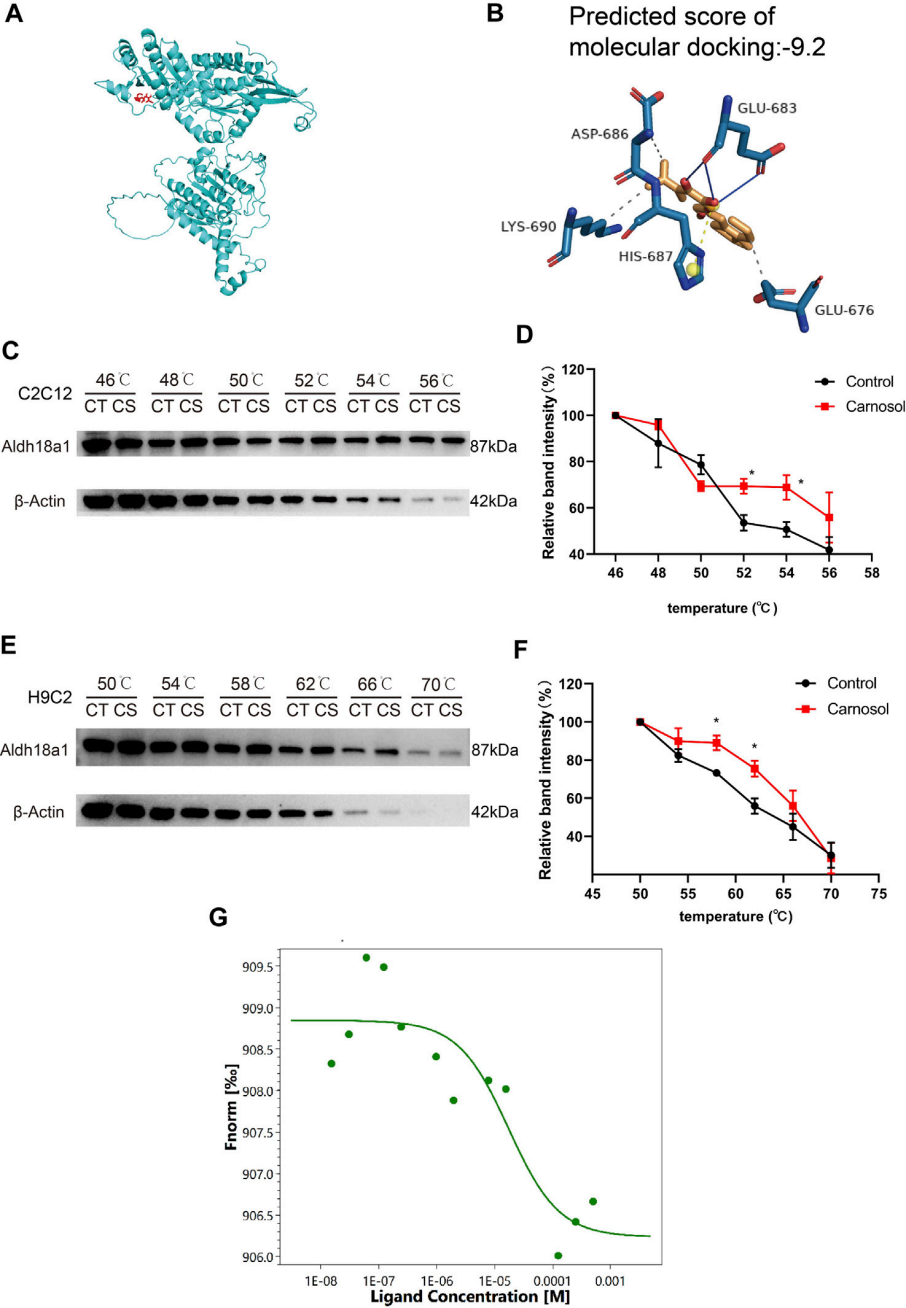
P5CS (Aldh18a1) might be the direct target protein of carnosol. **(A)** A representative image of binding between carnosol and P5CS. **(B)** The modified binding site and predicted score of protein-ligand interaction between carnosol and P5CS. **(C)** The representative results of CETSA assay in C2C12 cells. **(D)** The statistical result of CETSA assay in C2C12 cells. **(E)** The representative results of CETSA assay in H9c2 cells. **(F)** The statistical result of CETSA assay in H9c2 cells. **(G)** The result of MST assay. Data are presented as mean ± SEM (*n* = 3). *p < 0.05 vs. control group.

The DARTS/MS target finding system was conducted to find out the possible binding protein of carnosol. In the Table 1, P5CS possessed 5 unique peptides, and the ratio value of average abundance in the carnosol group/average abundance in the DMSO group was 1.3145, and the *p-value* is 0.0351, which has a high level of confidence. Results of molecular docking analysis showed that the predicted score of protein-ligand interaction binding between carnosol and P5CS was −9, which also suggested carnosol has an affinity to P5CS (Figures 5A, B). CETSA was employed to verify the binding between carnosol and P5CS, which is an experiment to study the target of drugs on the strength of examining the thermal stability of the proteins when the ligand binds. As shown in Figures 5C, D, the thermal stability of P5CS in C2C12 was improved after the incubation of myotubes with carnosol. Compared with control group, the level of P5CS was increased in C2C12 myotubes treated with carnosol. Furthermore, CETSA analysis using H9c2 cardiomyocytes also showed similar results that carnosol increased the thermal stability of P5CS (Figures 5E, F). Meanwhile, MST assay (Figure 5G) shows that carnosol could dose-dependently bind with P5CS, and the Kd value is 17.4 μM. The results of DARTS, CETSA and MST assay suggested the binding between P5CS and carnosol in C2C12 myotubes.

**TABLE 1:** The list of the possible direct target proteins of carnosol found in DARTS analysis.

Protein names	Accession ID	# Unique peptides	Average abundances (Solvent control)	Average abundances (CS)	Ratio average (CS)/Average (Solvent control)	*p*-value (CS vs. Solvent control)
Aldh18a1	Q9Z110	5	213.05	280.05	1.3145	0.0351	
Ifitm3	Q9CQW9	2	1,992.35	1,452.55	0.7291	0.0070	
Pycard	Q9EPB4	2	985.25	567.9	0.5764	0.0045	
Xpo5	Q924C1	2	61.2	82.05	1.3407	0.0183	
Dnm1l	Q8K1M6	2	71.7	55.2	0.7699	0.0116	
Edc3	Q8K2D3	2	58.2	84.9	1.4588	0.0101	
Chmp4b	Q9D8B3	2	89.1	123.05	1.3810	0.0211	
Atox1	O08997	1	607.2	406.4	0.6693	0.0093	
Mtm1	Q9Z2C5	1	60	79.5	1.3250	0.0376	
Ankzf1	J3QM81	1	3.95	5.55	1.4051	0.0171	
Cnot10	Q8BH15	1	39.4	53.85	1.3668	0.0400	
Zfp947	Q8BIQ6	1	21.6	47.8	2.2130	0.0294	
Luc7l3	Q5SUF2	1	55.7	74.25	1.3330	0.0383	
Dnase2b	Q9QY48	1	98.35	69.45	0.7062	0.0050	

### 3.6 Interaction network including direct target protein P5CS and indirect target proteins of carnosol

**FIGURE 6:**
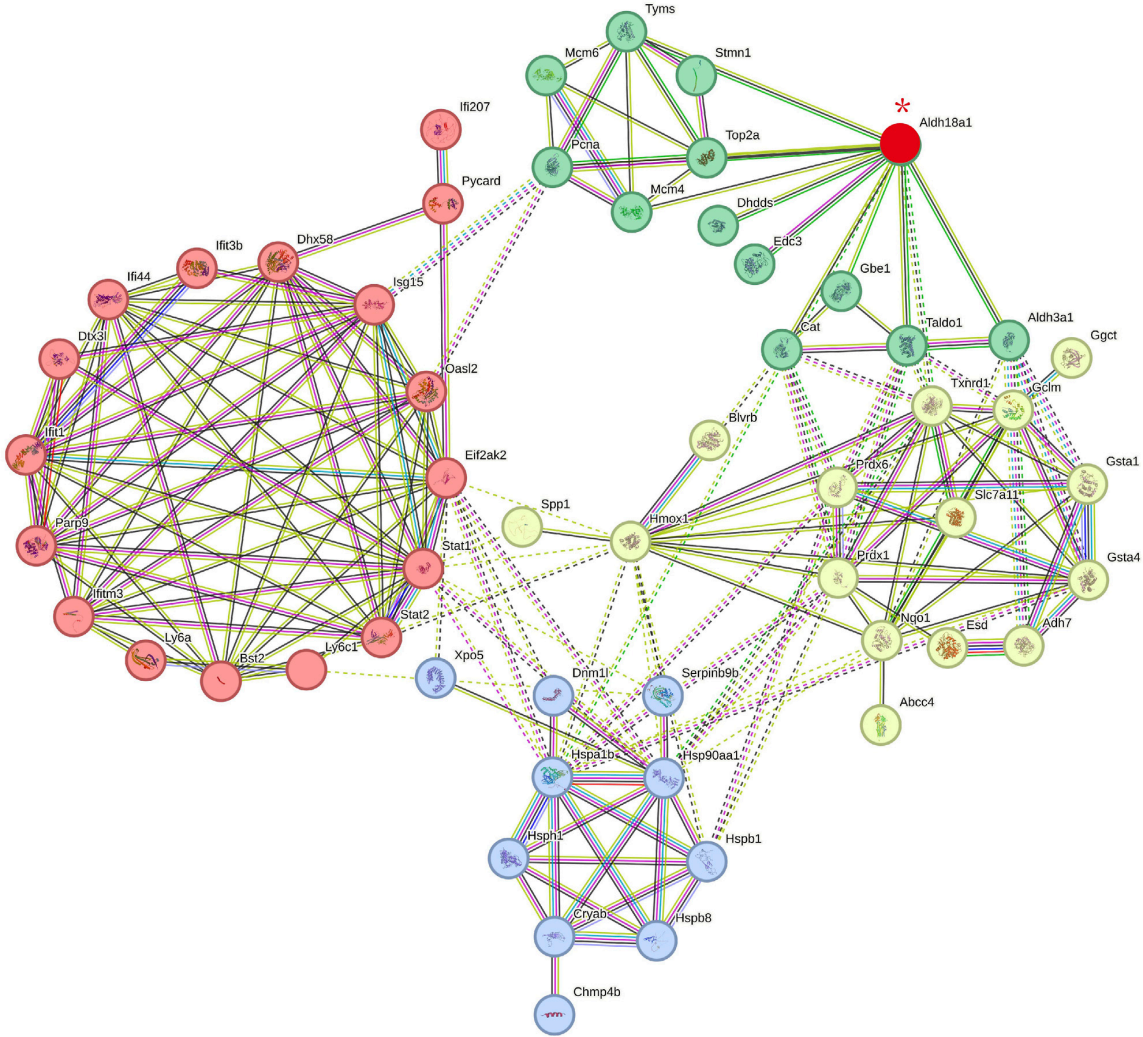
The protein-protein interaction network includes P5CS (Aldh18a1) (the direct target protein of carnosol) and the possible indirect target proteins of carnosol (the differentially expressed proteins in the C26+CS group compared with the C26 group). Proteins marked in red and with a red asterisk is P5CS (Aldh18a1). Proteins marked in green are related to catalytical system. Proteins marked in light yellow are related to glutathione metabolisms and antioxidant system. Proteins marked in blue are related to heat shock response. Proteins marked in red are related to immune and inflammation system. Edges represent protein-protein associations, in which sky-blue lines and pink lines are known interactions. Blue lines represent the evidence of associations are from curated databases while pink lines were experimentally determined. Green lines, red lines and blue lines represent predicted interactions, which represent gene neighborhood, gene fusions and gene co-occurrence respectively. Yellow-green, black, and purple lines represent associations of text meaning, co-expression and protein homology. Dotted lines represent associations between proteins in different clusters. The interaction score is provided in Supplementary Files.

To investigate the relationship between P5CS (Aldh18a1), the direct target protein of carnosol, and other indirect target proteins of carnosol, the string protein-protein interaction network including Aldh18a1 and the differentially expressed protein (DEPs) in the C26+CS group vs. C26 group with fold change >1.5 or fold change <0.5 was constructed. As shown in Figure 6, in the network, Aldh18a1 (P5CS, the protein marked in red and with a red asterisk) exhibited interaction mainly with three subsets of proteins including proteins related to glutathione metabolism and antioxidant system (proteins marked in light yellow) such as Gclm, Slc7a11, Prdx6 and Gsta5, and proteins related to heat shock response (proteins marked in blue) such as Hsp90aa1 and Hspb1. P5CS is the key enzyme for the synthesis of proline from glutamate and thus plays a critical role in regulating amino acid metabolism and glutamate metabolism. These results suggested that binding of carnosol to P5CS might cause changes in the expression of glutathione metabolism-related proteins and thus affect the anti-oxidant system and heat shock response to protect the myotubes against the myotube atrophy induced by C26 tumor medium.

### 3.7 The influence of P5CS (Aldh18a1) knockdown on the myotube atrophy induced by C26 medium and the ameliorating effects of carnosol

**FIGURE 7:**
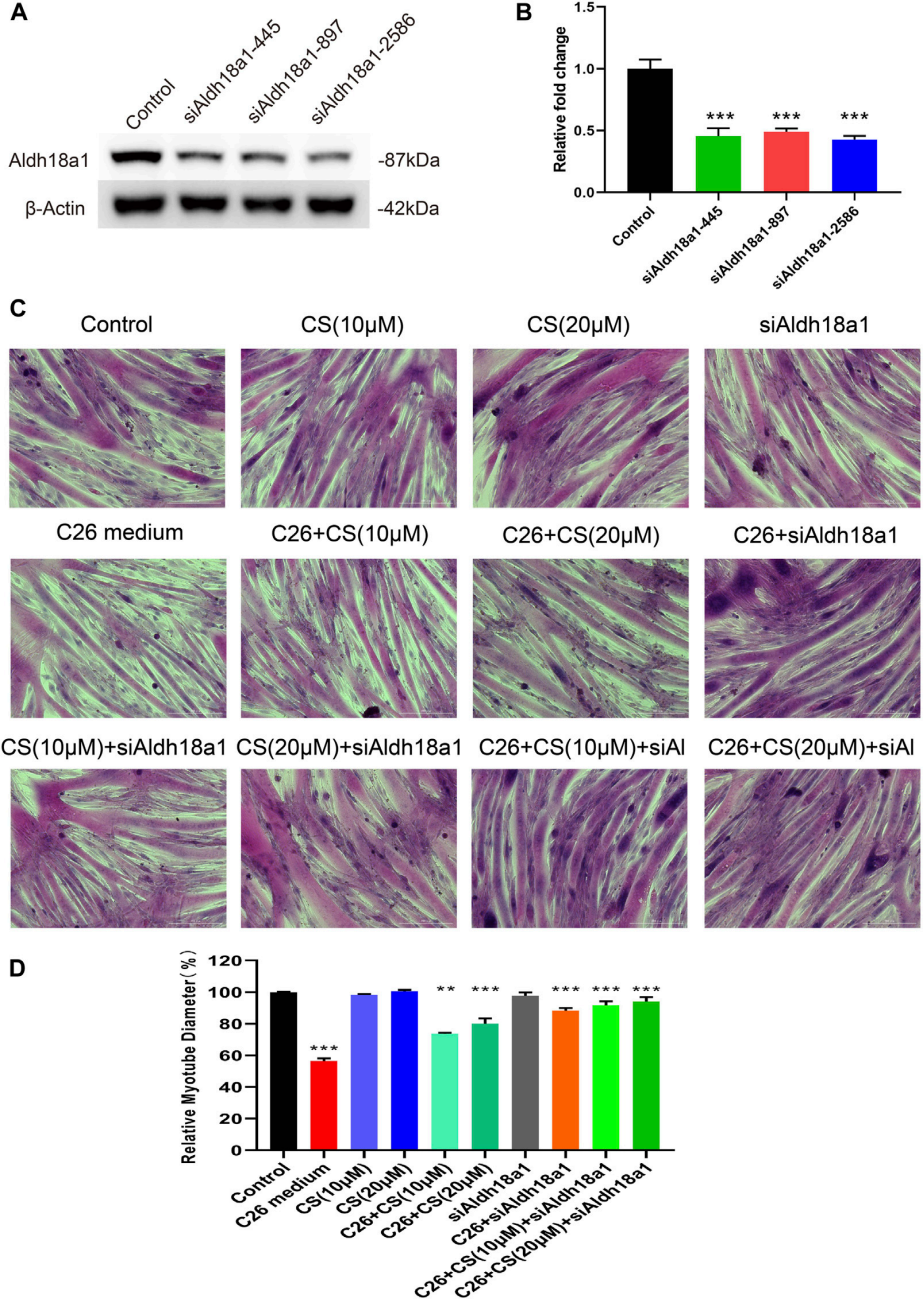
The influence of P5CS (Aldh18a1) knockdown on the myotube atrophy of C2C12 myotubes induced by C26 medium and the ameliorating effects of carnosol. **(A)** The Western blot verification of siRNA knockdown efficiency. **(B)** The results of the siRNA knockdown efficiency verification quantification. **(C)** Representative images of H&E staining of C2C12 myotubes underwent treatment of C26 medium with or without the presence of siAldh18a1 or carnosol. Scale bar, 200 μm. **(D)** The quantification results of myotube diameters in different groups. Data are presented as mean ± SEM (*n* = 3). *p < 0.05, **p < 0.01, ***p < 0.001 vs. control group; #*p* < 0.05, ##*p* < 0.01, ###*p* < 0.001 vs. C26 medium group.

As shown in Figure 7, to check the involvement of P5CS in C2C12 myotube atrophy and the alleviating effects of carnosol on it, siRNA for the gene encoding P5CS, siAldh18a1, was applied to knock down the expression of P5CS and then the myotube atrophy of siAldh18a1-treated C2C12 myotubes, with the treatment of C26 medium, with or without the presence of carnosol, was observed. The knock down efficiency of siRNA of Aldh18a1 was higher than 50% (Figures 7A, B) and the siAldh18a1 2586 was applied in later experiments. Representative results (Figure 7C) and the statistical results (Figure 7D) showed that, P5CS knockdown alone could effectively ameliorate myotube atrophy of C2C12 myotubes induced by C26 medium and, furthermore, the combination of P5CS knockdown with carnosol could further enhance the alleviating effects of carnosol. These results suggested the important role of P5CS in regulating myotube atrophy and the possible contribution of binding with P5CS to the effects of carnosol on myotube atrophy.

## 4 Discussion

In the present study, the ameliorating effects of carnosol on myotube atrophy induced by simulated cancer cachexia injury, conditioned medium of C26 tumor cells or LLC tumor cells, were checked and then the possible target proteins of carnosol in ameliorating cancer cachexia-related myotube atrophy were searched. C2C12 myotubes treated with the conditioned medium of C26 tumor cells or LLC tumor cells exhibited myotube atrophy (decrease in myotube diameter) while carnosol could significantly ameliorate the myotube atrophy induced by C26 medium or LLC medium. Proteomic analysis was used to check the protein expression profiles of C2C12 myotubes under the treatment of C26 medium with or without the presence of carnosol. Firstly, results of the proteomic analysis showed the activation of the NF-κB signaling pathway and cell adhesion signaling pathway under the C26 medium induction. Compared with the control group, C2C12 myotubes in the C26 medium-treated group exhibited upregulation of proteins related to the “NF-κB signal pathway,” “Cell adhesion molecules,” “ECM-receptor interaction,” etc. These results were consistent with previous reports about the muscle atrophy in cancer cachexia. NF-κB signaling is well known to play important roles in the initiation and progression of cancer cachexia muscle atrophy (Op den Kamp et al., 2013; Setiawan et al., 2023). Genes related to “Cell adhesion molecules” were enriched in the differentially expressed genes in comparing the gene expression profiles of clinical specimens of atrophic and normal muscle tissues (Mahmassani et al., 2019). Profiling of the muscle-specific dystroglycan interactome revealed the role of Hippo signaling, an important pathway in ECM-receptor interaction, in muscular dystrophy and age-dependent muscle atrophy (Yatsenko et al., 2020). To be noted, carnosol could partly reverse the upregulation of these proteins induced by C26 medium. For example, the expression of Tumor necrosis factor receptor superfamily member 6 (Fas, APT1, CD95/Apo-1) was increased in the C26 model group vs. control group while carnosol treatment resulted in the decreased expression of this protein in the C26+CS group compared with that of the C26 model group. Fas/Apo-1 is closely related to the regulation of the NF-κB signaling pathway and it was reported that Fas/Apo-1 could activate the NF-κB pathway (Rensing-Ehl et al., 1995; Wallach et al., 1999). The results suggested the activation of the NF-κB pathway in C2C12 myotubes under simulated cancer cachexia injury and the inhibition of carnosol on activation of the NF-κB pathway. Importantly, in our previous study, we also observed the inhibiting effects of carnosol on the NF-κB pathway activated in muscle tissues in cancer cachexia (Lu et al., 2021). The inhibitive effects of carnosol on the NF-κB pathway were also observed in other kinds of cells (Lo et al., 2002; Lian et al., 2010; Yao et al., 2014; Li et al., 2021). The regulating effects of carnosol on the expression levels of cell adhesion molecules had also been reported before (Yao et al., 2014). The effects of carnosol in regulating the expression of cell adhesion molecules were closely related to its inhibition on the NF-κB signaling pathway (Yao et al., 2014). Secondly, the results of the proteomic analysis showed the influence of carnosol on glutathione metabolism and the anti-oxidant system. Previous reports showed that the antioxidant activity of carnosol might be the basis of its inhibition on the NF-κB signaling pathway (Lian et al., 2010). The anti-oxidant activities of carnosol (Lian et al., 2010; Kalantar et al., 2021; Karagianni et al., 2022; Ji et al., 2023) and its regulation on glutathione metabolism (Singletary, 1996; Chen et al., 2011; Ishida et al., 2014; Samarghandian et al., 2017), which was closely related to the anti-oxidant system, had been reported in lots of studies. In the present study, carnosol treatment alone could increase the expression of proteins related to “Glutathione metabolism.” Furthermore, carnosol treatment under the induction of C26 medium also affected the expression of proteins related to the “Glutathione metabolic process,” “Glutathione metabolism,” “Anti-oxidant activity,” “Response to oxidative stress,” “Peroxidase activity,” “Oxidoreductase activity,” and “Cellular oxidant detoxification,” etc. These results clearly showed the influence of carnosol on glutathione metabolism and anti-oxidant system, which might be the basis of its ameliorating on muscle atrophy in cancer cachexia. Furthermore, the ROS level in the C26 medium group was abnormally elevated, whereas it was dramatically reduced in the C26+CS groups. These findings suggest that the carnosol treated groups were the recipients of the antioxidant effect.

Most importantly, P5CS was found in the present study to be the direct target protein of carnosol in C2C12 myotubes by using DARTS analysis. Totally 9 possible direct target proteins were found in our DARTS analysis. Among them, P5CS was the one with the highest credibility of 5 unique peptides in protein identification. Therefore, we concentrated on the study of P5CS in the present study. The possibility that carnosol might also bind proteins other than P5CS in C2C12 myotubes could not be excluded. P5CS has an important function for the regulation of glutamine metabolism and the Krebs cycle. The most important roles it plays are catalyzing the reduction conversion and coupled phosphorylation and synthesizing proline from glutamate (Guo et al., 2020; Kalmar et al., 2021; Pickwick et al., 2022). Furthermore, a recent study conducting a functional assessment of homozygous P5CS variants revealed alterations in amino acid and antioxidant metabolism (Colonna et al., 2023), which further supported the important role of P5CS in regulating the anti-oxidant system. Interestingly, derangements of amino acids in cachectic skeletal muscle such as significantly reduced quantities of glutamate were observed in both cancer cachexia animals as well as human cancer cachexia patients (Kunzke et al., 2020). These results suggested that amino acid metabolism dysregulation might also be involved in the development of muscle wasting in cancer cachexia. In the present study, the possible binding between P5CS (Aldh18a1) and carnosol was found in the DARTS analysis and then confirmed by CETSA assay and MST assay. Finding P5CS (Aldh18a1) as the direct target of carnosol provided explanations for the regulating effects of carnosol on glutathione metabolism (Singletary, 1996; Chen et al., 2011; Ishida et al., 2014; Samarghandian et al., 2017). In the present study, the influence of carnosol on the amino acid profiles in the C2C12 myotubes was checked. Importantly, carnosol treatment could significantly increase the level of glutamine in C2C12 myotubes. C26 medium induced a decrease in the glutamine level while carnosol could partly ameliorate the decrease of glutamine induced by the C26 medium. Glutamine could be transformed into glutamate, which plays a role in regulating the synthesis of glutathione (Tapiero et al., 2002; Park and Kim, 2020). Increasing glutathione in skeletal muscle could enhance cell growth, surface antigen expression, and heat shock protein synthesis (Roth et al., 2002). Furthermore, carnosol treatment could also ameliorate the decrease in other amino acids such as asparagine, leucine, isoleucine, and valine induced by the C26 medium. The increase in amino acid levels might contribute to an increase in protein synthesis. For example, supplementing with glutamine and arginine might enhance protein synthesis and reverse cancer-related wasting (May et al., 2002). Supplementing with leucine + isoleucine + valine or glutamine + arginine could help reduce oxidative stress and cellular energy imbalance (Ahmed R.F.et al., 2022). Our results of the amino acid profile analysis showed that carnosol had a considerable influence on amino acid metabolism, especially that of glutamine, and related glutathione metabolism. At the same time, our results of the proteomic analysis also showed that, carnosol treatment induced change in the expression of proteins related to “Glutathione metabolism” as well as “Valine, leucine and isoleucine degradation,” “Glycine, serine and threonine metabolism,” “Lysine degradation,” etc. The influence of carnosol on amino acid metabolism, especially glutamine metabolism and glutathione metabolism, might contribute to the ameliorating effects of carnosol on muscle atrophy in cancer cachexia.

Based on the direct target protein of carnosol (P5CS) and the in-direct target proteins of carnosol (differentially expressed proteins found in the C26+CS group compared with that of the C26 medium group), the signal network of carnosol was constructed. In the network, three subsets of proteins including 1) proteins related to glutathione metabolism, 2) proteins related to the antioxidant system, and 3) proteins related to heat shock response were found to have close interaction with P5CS. Firstly, proteins related to glutathione metabolism such as Slc7a11, Txnrd1, Nqo1, Gclm, etc. Were found to be significantly upregulated while proteins such as Ggct were found to be significantly downregulated under carnosol treatment. Cysteine is the rate-limiting component of glutathione biosynthesis and it can be transported into the cell via neutral amino acid transporters. Slc7a11 (cystine/glutamate antiporter solute carrier family 7 member 11), also known as xCT, is the light chain subunit of cystine/glutamate antiporter system xc−, which functions as a cystine/glutamate antiporter to import one molecule of cystine in exchange for one molecule of intracellular glutamate (Koppula et al., 2018). Txnrd1 (cytosolic thioredoxin reductase) is a selenocysteine-containing oxidoreductase flavoprotein which is a component of the mammalian thioredoxin system (Bjorkhem-Bergman et al., 2014). The metabolism of quinones and glutathione is remarkably intertwined and Nqo1 [NAD(P)H dehydrogenase (quinone) 1] is the prime cytosolic quinone reductase (Watanabe et al., 2004). Gclm (Glutamate-cysteine ligase regulatory subunit) is the modifier subunit of the glutamate-cysteine ligase, the rate-limiting enzyme of the synthesis of glutathione (Mohar et al., 2009). Ggct (Gamma-glutamylcyclotransferase) catalyzes the formation of 5-oxoproline from gamma-glutamyl dipeptides and plays a significant role in glutathione homeostasis (Oakley et al., 2008). Secondly, proteins related to the anti-oxidant system such as Gsta5, Gsta1, Gsta4, Prdx6, Prdx1, etc. Were found to be significantly upregulated by carnosol treatment. Glutathione-S transferases (GSTs) including Gsta5, Gsta1, and Gsta4 are the major contributors to the eukaryotic cell’s defense against chemical and oxidative stress (Kazi and Ellis, 2002). Peroxiredoxins (Prxs) including Prdx6, Prdx1 are enzymes that exert antioxidant function and eliminate ROS within cells, thus prevent damages caused by oxidative stress (Kim and Jang, 2019). Thirdly, proteins related to heat shock response such as Hsp90aa1 (Heat shock protein HSP 90-alpha), Hspb1 (Heat shock protein beta-1), Hspa1b (Heat shock 70 kDa protein 1B), Cryab (Alpha-crystallin B chain), etc. Were found to be significantly upregulated under carnosol treatment. Interestingly, the Hsp90 protein had been reported to be the target protein of carnosol (Shi et al., 2020). In mouse bone marrow-derived macrophages, carnosol plays an important part in inhibiting NLRP3 inflammasome activation through binding with Hsp90, and inhibiting its ATPase function (Shi et al., 2020). In the present study, though Hsp90 was not identified as the direct target protein of carnosol in the C2C12 myotubes, a series of heat shock proteins including Hsp90aa1 were found to be the indirect target proteins of carnosol. Our results suggested the important role of regulation on heat shock response in the mechanisms of carnosol. To be noted, the three subsets of proteins regulated by carnosol were closely related. In mammalian cells, glutathione may assist antioxidant enzymes such as glutathione peroxidases to clean out the ROS, though it is just a nonprotein sulfhydryl compound. Furthermore, the change in oxidative stress and redox state would induce the change in the expression of proteins related to glutathione metabolism as well as proteins related to heat shock response. Therefore, proteins related to glutathione metabolism, proteins related to the antioxidant system, and proteins related to heat shock stress have close interactions. Under carnosol treatment, the cooperation of the three subsets of proteins might be important in keeping cellular oxidative and redox homeostasis, inhibiting inflammatory pathways and thus protecting the cells from injuries such as cancer cachexia injury.

In summary, the results of the present study suggested that the direct target protein of carnosol in myotubes is P5CS while the indirect target proteins of carnosol might mainly include proteins related to glutathione metabolism, antioxidant system, and heat shock response. By binding with P5CS and regulating the amino acid levels and the expression of proteins related to glutathione metabolism, antioxidant system, and heat shock response, carnosol could protect myotubes against cancer cachexia injury. Notably, the knockdown of P5CS by siRNA in C2C12 myotubes also could significantly ameliorate myotube atrophy induced by the C26 medium. Furthermore, the combination of Aldh18a1 siRNA and carnosol could further efficiently protect myotubes against myotube atrophy induced by the C26 medium. These results confirmed the involvement of P5CS in the effects of carnosol in ameliorating cancer cachexia-like myotube atrophy. However, the mechanism by which carnosol treats cachexia is not well understood, and further studies are needed to be performed. It would be better to know whether the drug binding to the target Aldh18a1 has any effect on its enzyme activity and how its downstream protein pathway is regulated. Early *in vivo* experiments and *in vitro* cellular activity and cytotoxicity studies of carnosol (Lu et al., 2021) exhibited the safety of carnosol, since there is no toxic response at 10 mg/kg in mouse cancer cachexia model. And in the present study, the 20 μM carnosol exerted best ameliorating effect, and it kept on exerting anti-cachexia effect for 72 h while no apparent toxicity was demonstrated, thus long-term use of carnosol for cancer cachexia could be acceptable.

## 5 Conclusion

Carnosol could bind with P5CS, regulate the amino acid metabolism, glutathione metabolism, antioxidant system, and heat shock response, and thus exhibit protecting effects on myotubes from myotube atrophy induced by cancer cachexia injury.

## Data Availability

The datasets presented in this study can be found in online repositories. The names of the repository/repositories and accession number(s) can be found in the article/Supplementary Material.

## Supplementary material

SUPPLEMENTARY TABLE S1 Excel1-DARTSMS-potential targets of CS based on protein abundant.

SUPPLEMENTARY TABLE S2 Excel2-DARTSMS-potential targets of CS based on peptides abundant.

SUPPLEMENTARY TABLE S3 Proteome result1-C26_vs_CT_diff. detail.

SUPPLEMENTARY TABLE S4 Proteome result2-C26_CS_vs_C26_diff. detail (Fan et al., 2022).
