# Supplementary material for: Carnosol ameliorated cancer cachexia-associated myotube atrophy by targeting P5CS and its downstream pathways
Source: Front Pharmacol. 2024 Jan 5;14:1291194. doi: 10.3389/fphar.2023.1291194 (PMC10799341; doi:10.3389/fphar.2023.1291194)
Supplement: Supplementary file 5 [file DataSheet1.docx]

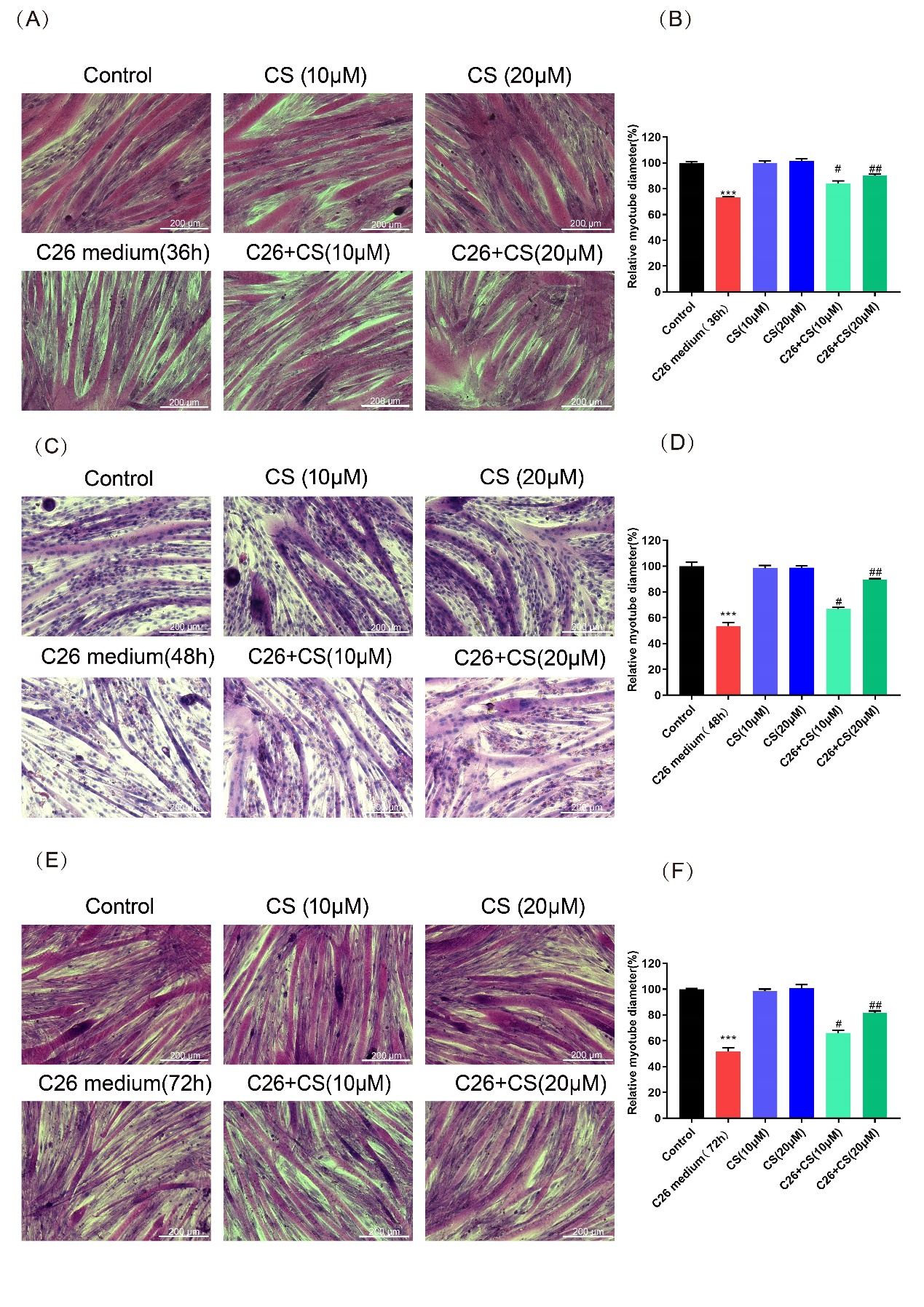


**Figure S1.** Carnosol ameliorated the myotube atrophy of C2C12 myotubes induced by the C26 tumor medium for different time periods (36, 48 or 72 h). (A) Representative images of H&E staining of C2C12 myotubes underwent treatment of C26 medium for 36 h with or without the presence of carnosol (CS). Scale bar, 200 μm. (B) The quantification results of myotube diameters in different groups after 36h-treatment. (C) Representative images of H&E staining of C2C12 myotubes underwent treatment of C26 medium for 48 h with or without the presence of carnosol (CS). Scale bar, 200 μm. (D) The quantification results of myotube diameters in different groups after 48h-treatment. (E) Representative images of H&E staining of C2C12 myotubes underwent treatment of C26 medium for 72 h with or without the presence of carnosol (CS). Scale bar, 200 μm. (F) The quantification results of myotube diameters in different groups after 72h-treatment. Data are presented as mean ± SEM (n = 3). ***p < 0.001 vs. control group; #p< 0.05, ##p < 0.01 vs. C26 medium group.


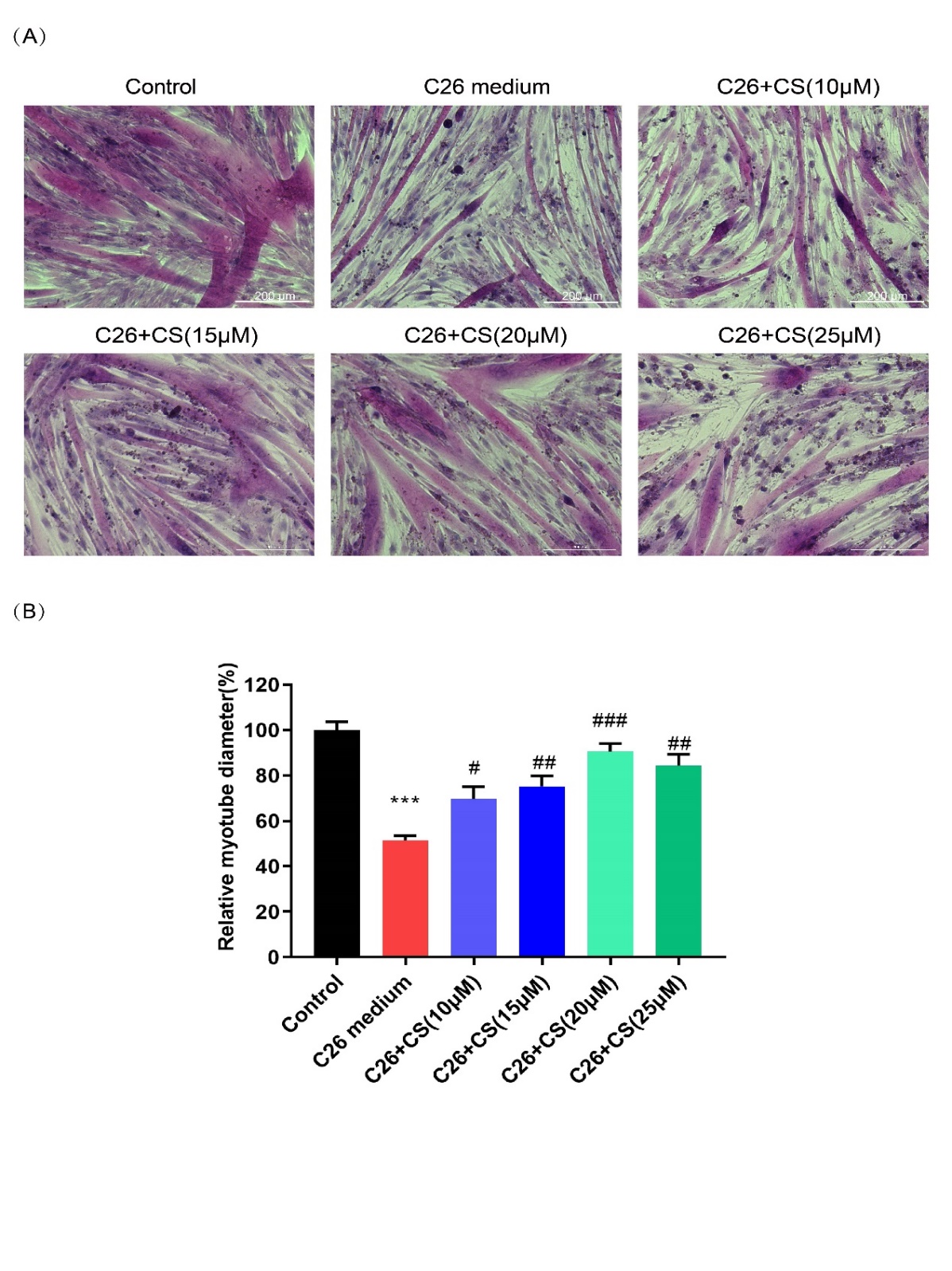


**Figure S2.** Carnosol at different doses ameliorated the myotube atrophy of C2C12 myotubes induced by the C26 tumor medium. (A) Representative images of H&E staining of C2C12 myotubes underwent treatment of C26 medium with the presence of different concentrations of carnosol (CS). Scale bar, 200 μm. (B) The quantification results of myotube diameters in different groups. Data are presented as mean ± SEM (n = 3). ***p < 0.001 vs. control group; #p< 0.05, ##p < 0.01, ###p < 0.001vs. C26 medium group.
